# Exploring Pharmacological Mechanisms of Essential Oils on the Central Nervous System

**DOI:** 10.3390/plants11010021

**Published:** 2021-12-22

**Authors:** Giselle A. Borges e Soares, Tanima Bhattacharya, Tulika Chakrabarti, Priti Tagde, Simona Cavalu

**Affiliations:** 1 Department of Medicinal and Biological Chemistry, University of Toledo, 3000 Arlington Ave., Toledo, OH 43614, USA; giselleamanda.borgesesoares@rockets.utoledo.edu; 2 Innovation, Incubation & Industry (I-Cube) Laboratory, Techno India NJR Institute of Technology, Udaipur 313003, Rajasthan, India; 3 Department of Science & Engineering, Novel Global Community Educational Foundation, Hebersham, NSW 2770, Australia; 4 Department of Chemistry, Sir Padampat Singhania University, Udaipur 313601, Rajasthan, India; tulika.chakrabarti@spsu.ac.in; 5 Bhabha Pharmacy Research Institute, Bhabha University Bhopal, Bhopal 462026, Madhya Pradesh, India; tagde_priti@rediffmail.com; 6 PRISAL Foundation (Pharmaceutical Royal International Society), Bhopal 462042, India; 7 Faculty of Medicine and Pharmacy, University of Oradea, P-ta 1 Decembrie 10, 410087 Oradea, Romania

**Keywords:** essential oils, aromatherapy, CNS, nanomedicine, Alzheimer’s disease

## Abstract

Essential oils (EOs) have been traditionally used as ancient remedies to treat many health disorders due to their enormous biological activities. As mainstream allopathic medication currently used for CNS disorders is associated with adverse effects, the search to obtain safer alternatives as compared to the currently marketed therapies is of tremendous significance. Research conducted suggests that concurrent utilization of allopathic medicines and EOs is synergistically beneficial. Due to their inability to show untoward effects, various scientists have tried to elucidate the pharmacological mechanisms by which these oils exert beneficial effects on the CNS. In this regard, our review aims to improve the understanding of EOs’ biological activity on the CNS and to highlight the significance of the utilization of EOs in neuronal disorders, thereby improving patient acceptability of EOs as therapeutic agents. Through data compilation from library searches and electronic databases such as PubMed, Google Scholar, etc., recent preclinical and clinical data, routes of administration, and the required or maximal dosage for the observation of beneficial effects are addressed. We have also highlighted the challenges that require attention for further improving patient compliance, research gaps, and the development of EO-based nanomedicine for targeted therapy and pharmacotherapy.

## 1. Introduction

**Figure 1:**
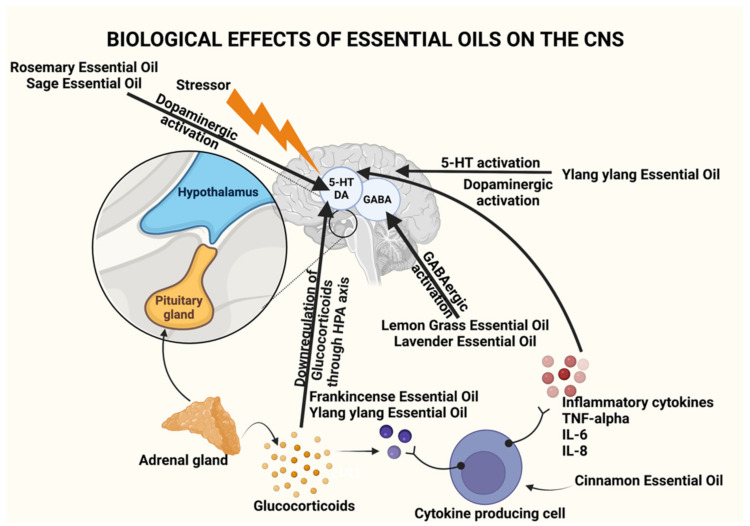
Biological effects of essential oils on the CNS through activation of various components of the brain. Created using Biorender.com. (Last accessed on 14 December 2021).

Since ancient times, essential oils (EOs) have been widely used and have been identified as therapeutic agents owing to their pharmacological and psychological properties. They were deemed to be physical, spiritual, and mental healing agents [1,2]. EOs are naturally occurring complex mixtures of volatile odor compounds synthesized as secondary metabolites by plants and are extracted through steam distillation, solvent extraction, maceration, cold press extraction, water distillation, and CO_2_ extraction. Novel methods that are more efficient and provide higher yields include supercritical fluid extraction, microwave-assisted extraction, and ultrasound [3]. Studies conducted on animals and humans have shown that EOs can produce a variety of CNS targeted pharmacological effects such as anxiolytic effect, neuroprotection, antidepressant effect, anticonvulsant effect, analgesic, and sedative effect, to name a few. As a result, EOs can be used as an adjuvant therapy to prevent and relieve symptoms associated with CNS-based disorders such as insomnia, depression, dementia, Alzheimer’s disease (AD), etc. As they are naturally occurring, they have the added benefit of being non-toxic and safe when utilized correctly at appropriate concentrations, which have been proven through research in the last ten years [2]. Table 1 provides a summary of the source, active constituents, and methods of extraction of EOs, and Table 2 provides a summary of the dosage, preclinical and clinical studies pertaining to the use of EOs, while Figure 1 illustrates the various mechanisms by which EOs have been found to act on the CNS.

The term ‘aromatherapy’ was coined in the early 20th century by Rene M. Gattefosse. In his book published in 1973 titled ‘Aromatherapie,’ he claimed that he could cure any ailment of the human body through herbal medicines [66]. Although the benefits remain controversial, patients and healthcare providers are highly attracted to EOs use due to their low cost and lesser potential to show adverse effects. The therapeutic effects of EOs have been observed following topical application or inhalation. The topical application involves adding a few drops of EO to carrier oil such as olive oil, coconut oil, argan oil, etc., followed by massaging the skin or area of interest to promote absorption through pores and hair follicles. Inhalation therapy involves using a diffuser, humidifier, or soaking gauze with EO, which is then kept near the patient for inhalation. Inhalation of EOs causes stimulation of olfactory nerves, which are specialized sensory nerves responsible for the sense of smell [67].

According to a review paper by Yang et al., the parts of the brain associated with pain perception include the primary somatosensory cortex, secondary somatosensory cortex, anterior cingulate cortex (ACC), prefrontal cortex (PFC), insular cortex, amygdala, thalamus, cerebellum, and periaqueductal gray (PAG) [68]. Therefore, researchers hypothesized that the analgesic effects associated with certain essential oils could be attributed to targeting certain regions of the brain. In 2018, Ze-Jun Wang et al. reviewed mechanisms by which essential oils exerted their antinociceptive, anxiolytic, and anticonvulsant effects. They suggested that these effects are due to essential oils’ ability to primarily modulate the GABAergic system and sodium (Na^+^) ion channels and the capability to target transient receptor potential (TRP) channels [69].

According to the WHO, worldwide, more than 264 million people are affected by depression, and approximately 800,000 people die due to suicide each year [70]. Currently, therapies include behavioral activation, cognitive behavioral therapy (CBT), interpersonal psychotherapy, and antidepressant medication such as selective serotonin/serotonin-norepinephrine reuptake inhibitors (SSRIs) and tricyclic antidepressants (TCAs) [2,8]. Although SSRIs have shown benefit/risk balance [71], currently administered medications for depression are associated with significant side effects and lead to sub-optimal therapeutic outcomes in some patients. Ogata et al. performed a study on healthy males to determine the mechanism by which lavender oil exerts its mood-elevating effect. Lavender oil was inhaled for 20 min, followed by subsequent inhalation a week later. The study concluded that lavender oil offers therapeutic benefits for stress relief owing to its ability to activate the central oxytocin neurons [72].

The most common form of dementia is that of Alzheimer’s disease. The WHO has estimated that currently worldwide, 50 million people are affected and has projected that in 2030 and 2050, the number of cases will increase to 82 million and 152 million, respectively. Currently, there is no treatment available to cure and prevent the progression of dementia. However, cholinesterase inhibitors like donepezil, rivastigmine galantamine, and an N-methyl-d-aspartate (NMDA) antagonist memantine have been used to reduce and control behavioral symptoms [73,74]. Observed side effects associated with SSRIs and TCAs include weight gain, headaches, tachycardia, and sexual dysfunction [75]. Filipstova et al. measured the ability of rosemary oil and lavender oil to retain the short-term image and numerical memory. The result of this study indicated that EOs do exert their CNS effects through varying mechanisms and target different regions and receptors of the brain [76].

All the available information presented in the paper was compiled and acquired from library searches and electronic databases such as PubMed, Google Scholar, Science Direct, and Web of Science. Factual statistical information was obtained from web pages the WHO, NIH, and Mayo Clinic web pages, to name a few. The main purpose of this review paper is to establish the significance and mechanism of action of EOs as a complementary and adjuvant therapy to alleviate and relieve symptoms associated with CNS based disorders, to highlight domains that require further research, and to address the significance and need to develop patient compliant EO-based NANOMEDICINEs for targeted therapy or pharmacotherapy.

## 2. Methodology

**Figure 2:**
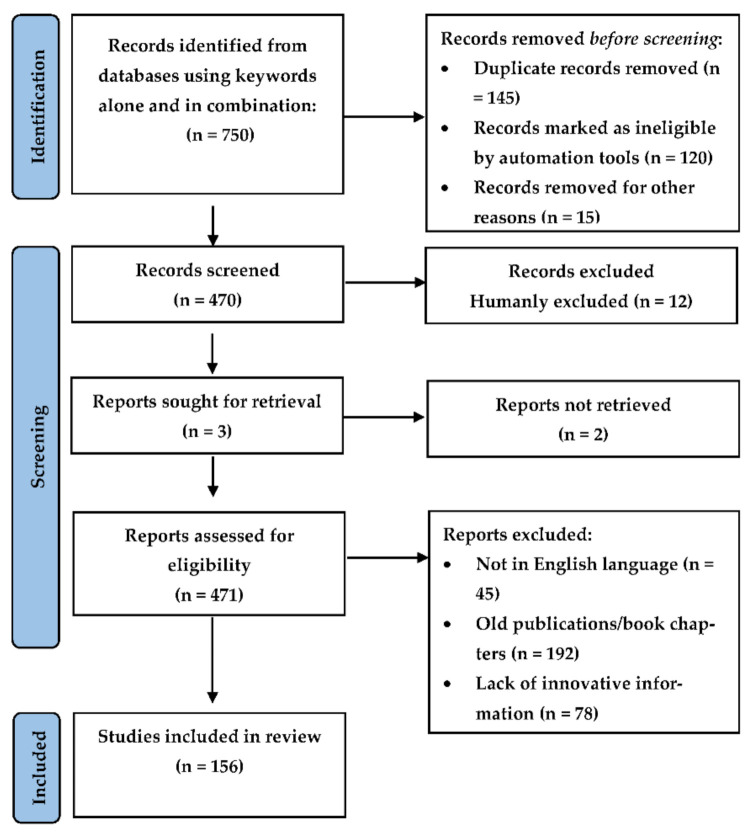
Flow chart presenting the selection protocol followed for the inclusion of published data into the present paper.

Relevant studies pertaining to the biological activity, mechanism of action at the molecular level, and the neuropharmacology of EOs involved in providing beneficial effects in CNS-based disorders were selected through the application of an algorithm (based on the recommendations of Page et al. [77,78]) that is presented as a flow-chart in Figure 2, indicating the steps/selection criteria followed to obtain necessary material for our review. Literature was obtained from several scientific databases such as PubMed (http://www.ncbi.nlm.nih.gov/pubmed), Google Scholar (http://www.scholar.google.co.in), Elsevier (https://www.elsevier.com/en-in), Science Direct (http://www.sciencedirect.com), Wiley (http://www.onlinelibrary.wiley.com), Springer Link (http://www.springer.co.in), and Scopus (http://www.scopus.com) (Last accessed on 14 December 2021). Literature was also obtained from book chapters and conference proceedings. The search of the different plants, their components, and techniques of extraction was performed by using the keywords such as Syzygium aromaticum, Boswellia sp., lavender oil, Lavandula angustifolia, Cymbopogon citratus, lemongrass oil, Cananga odorata, Ylang Ylang oil, cinnamon oil, Cinnamomum verum, cinnamon essential oil, Eucalyptus globulus, Eucalyptus oil, Mentha piperita, peppermint oil, Rosmarinus officinalis, rosemary oil, Salvia sclarea, sage oil, Santalum paniculatum, sandalwood oil, CNS effects of essential oils, animal testing, molecular mechanism of essential oils, anxiolytic, antidepressant, oxidative, analgesic effects. Scientific names of plants were obtained through Wikipedia (https://en.wikipedia.org/wiki/) and confirmed from The Plant List (http://www.theplantlist.org/) (Last accessed on 12 December 2021). Statistical data were obtained from webpages of the WHO, NIH, and MayoClinic. Preclinical and clinical data between 2003 and 2021 were included in this review. Only publications and book chapters restricted to the English language were reviewed. ChemDraw Professional 20.1.0 was used to authenticate chemical names and to draw figures of chemical structures. Other figures were created using Biorender.com (Last accessed on 14 December 2021).

## 3. Therapeutic Benefits of Essential Oils

**Figure 3:**
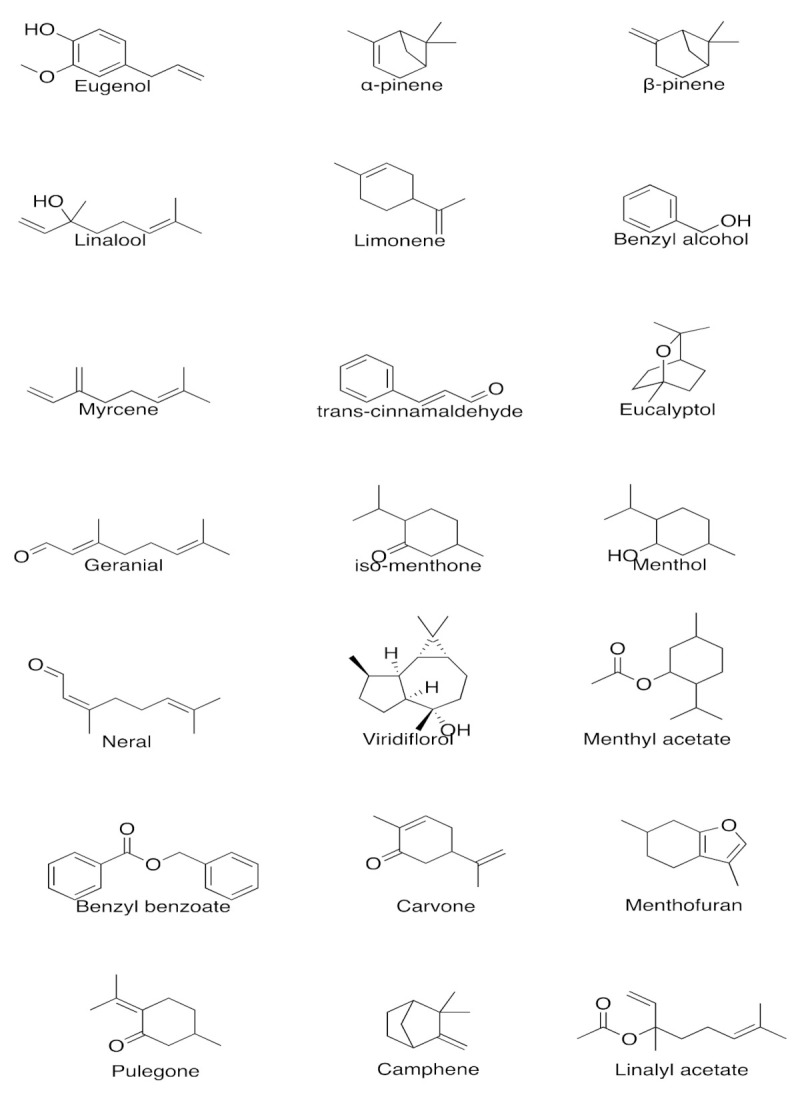
Chemical structures of bio-active constituents present in EOs acting on the CNS. Created using ChemDraw 20.1.0. URL: https://perkinelmerinformatics.com/products/research/chemdraw/ (accessed on 14 December 2021).

The effects and the mechanism of action of EOs are dependent upon their chemical composition, the molecular structure of the bioactive constituents, as well as the position and stereochemistry of the functional groups within the molecule [79]. EOs offer a multitude of benefits, which have been discussed in subsequent sections. Moreover, due to the large number of constituents present in oils, they possess a wide range of benefits and can be used for the treatment of various disease states. The chemical structures of the bio-active constituents that are described in this paper are illustrated in Figure 3.

### 3.1. Role in Pain Management

About 84% of old patients suffer chronic pain that is undiagnosable, persistent, and complex. This further leads to a reduction in the quality of life coupled with anxiety and poor sleep. Moreover, 70–85% of the geriatric population suffers from chronic back pain. With respect to women, 25–97% suffer from menstrual pain, while 15% of the female population suffer from severe pain causing impairment in day-to-day activities [67].

According to a review paper by Yang et al., the parts of the brain associated with pain perception include the primary somatosensory cortex, secondary somatosensory cortex, anterior cingulate cortex (ACC), prefrontal cortex (PFC), insular cortex, amygdala, thalamus, cerebellum, and periaqueductal gray (PAG) [68]. Therefore, researchers hypothesized that the analgesic effects associated with certain EOs could be attributed to targeting certain regions of the brain. The constituents that possess analgesic activity, as well as their mechanism of action, are discussed below.

- Clove Oil

The analgesic and anti-inflammatory effect of clove oil, especially for toothaches, is well documented. The main constituent of clove oil is eugenol, which is highly therapeutic [80,81]. Studies conducted by Chung et al. [82] show that the analgesic effects of eugenol occur through the inhibition of voltage-gated sodium (Na^+^) and Ca(V) 2.2, 2.3 calcium (Ca^2+^) channels and currents without the involvement of transient receptor potential cation channel vanilloid 1 (TRPV1). Eugenol also causes the inhibition of pro-inflammatory mediators such as lipoxygenase, interleukin 1β, cyclo-oxygenase, and nitric oxide synthase [83]. Studies conducted by Xu et al. determined that TRPV3, which is a heat sensitive Ca^2+^ permeable ion channel in the skin, tongue, and nose, is expressed by eugenol [5].

Following the entry into the bloodstream, either through inhalation or massage therapy, the analgesic activity of eugenol on the CNS is attributed to the ability of eugenol to potentiate the GABA_A_, receptors thereby increasing the affinity of GABA to the receptors, a mechanism observed in benzodiazepines and barbiturates [17,81,84]. Moreover, studies conducted by Bo et al. suggested that eugenol can modulate glutamatergic receptors and inhibits TNF-α [85].

In addition to being analgesic, eugenol is also associated with antioxidant and antidepressant activity, as confirmed by Dhiman et al. [86], who designed and synthesized eugenol-based derivatives, performed in vitro, in silico studies, and tested their MAO (A and B) inhibitory activity as agents for neurological disorders. Radical scavenging activity was also determined using H_2_O_2_ and DPPH scavenging methods followed by spectrophotometric titrations. All the synthesized compounds showed significant MAO inhibition through interaction with the MAO active site, as observed through molecular docking studies. Two synthesized compounds showed activity hMAO-A inhibition with IC_50_ values of 5.989 ± 0.007 µM and 7.348 ± 0.027 µM with a selectivity index of 0.19 and 0.14, respectively, while two other synthesized compounds showed hMAO-B inhibitory activity with IC_50_ values of 7.494 ± 0.014 µM and 9.183 ± 0.034 µM with a selectivity index of 5.14 and 5.72, respectively, indicating their potential antioxidant activity in antidepressant therapy and neurological disorders [86].

- Lavender Oil

**Figure 4:**
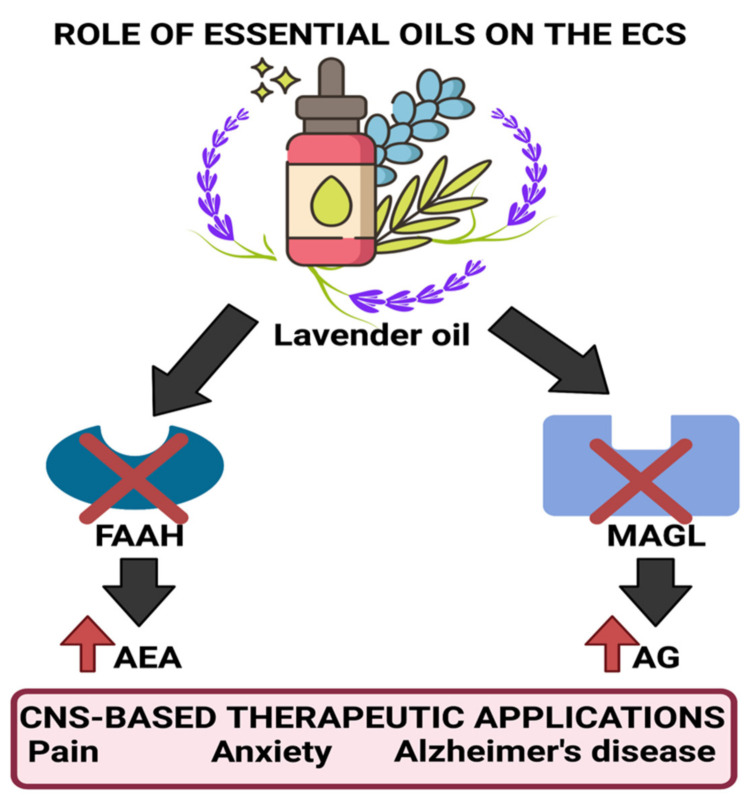
Ability of lavender oil to inhibit the degradation of FAAH and MAGL, thereby increasing levels of AEA and AG, which assist in mood elevation and analgesic effects. Created using Biorender.com. (Last accessed on 14 December 2021).

Due to many bio-active constituents, lavender oil can be used for various functions. According to studies conducted by Pinto et al., the analgesic effect of (-) linalool is attributed to the inhibition of the release of substance P or through antagonistic action on its receptor neurokinin-1 (NK-1) [87]. Moreover, (-) linalool also can cause inhibition of the active field potentials that occur through the antidromic stimulation of the hylus, indicating its ability to activate the voltage-gated Na^+^ channels in the granular neurons of the hippocampal dentate gyrus [88,89]. It also can modulate neurogenic and inflammatory pain through a reduction in peripheral and central nerve excitability [88]. (-) Linalool is also reported to cause a significant decrease in carrageenin-induced edema and acetic acid-induced writhing. This effect was diminished in the presence of atropine, a muscarinic receptor antagonist, and by naloxone, an opioid receptor antagonist indicating its cholinergic activity. Studies conducted by Peana et al. agree with the demonstrated pharmacological properties of linalool. They confirmed its ability to act as a cholinergic, local anesthetic and causes blockage of NMDA receptors. They also suggested that a key role in its activity is related to the opening of potassium (K^+)^ channels, which possibly occurs due to the stimulation of muscarinic M2, opioid, or dopamine D2 receptors [90,91]. Research conducted by Tashiro et al. on orexin neuron-deficient and orexin peptide-deficient mice subjected to formalin tests showed that orexinergic transmission was essential for linalool odor-induced analgesia, indicating that linalool caused the activation of hypothalamic orexin neurons, which act as critical mediators for processing pain [92]. Other studies conducted indicated that lavender oil was also found to bring about a decrease in ERK1, ERK2, and JNK1 phosphorylation along with iNOS level reduction. Moreover, lavender oil was also found to inhibit the degradation of FAAH (fatty acid amide hydrolase) and MAGL (monoacylglycerol lipase), thereby causing significant antinociception through the elevation of endocannabinoid levels in neuropathic pain models. These enzymes are essential for the synthesis and degradation of endocannabinoids as per the requirements of the body. Inhibition of FAAH and MAGL degradation causes the upregulation of AEA (anandamide) that has been found to be involved in emotion regulation. Figure 4 illustrates the process by which lavender oil exerts its effects on the endocannabinoid system (ECS) [93].

### 3.2. Role in Anxiety Relief and Stress Management

Anxiety disorders are the most common mental disorders in the United States, affecting approximately 40 million people aged 18 or older. They occur due to many reasons, such as genetics, life events, personality, and brain chemistry. Although treatable, only 36.9% of the population receive treatment. Anxiety disorders include generalized anxiety disorder (GAD), panic disorder (PD), social anxiety disorder, obsessive-compulsive disorder (OCD), stress, post-traumatic stress disorder (PTSD), major depressive disorder, and persistent depressive disorder (PDD) [94]. The current primary treatments for anxiety include psychotherapy such as cognitive-behavioral therapy (CBT), antidepressant drugs, and anti-anxiety medication such as buspirone, benzodiazepines, and ß-blockers, which are associated with a lot of side effects [95]. The essential oils that can be used for anxiety relief and stress reduction include:

- Frankincense oil

The essential oil of Frankincense contains 147 compounds that attribute to its activity, such as α-pinene, β-pinene, α-thujene, myrcene, sabinene, limonene, para cymene, and β-caryophyllene [7]. An animal model-based study conducted in 2019 by Okano et al. indicated a significant reduction in the levels of stress marker corticosterone and the endogenous antioxidant glutathione when administered in the undiluted and diluted form (1:1000) with jojoba oil, thus indicating the attenuation of induced stress by the essential oil of Frankincense. A decline in non-rapid eye movement and enhancement of wakefulness time was also observed after administering the diluted form. However, upon isolation of significant components, α-pinene and limonene from the oil, a decline in corticosterone levels was not observed, indicating that the constituents of the essential oil work synergistically to produce the anxiolytic effect [8].

- Lavender oil

As mentioned earlier, the major constituents of lavender oil are believed to exert their effects through interactions with the GABAergic system [11]. Concerning anxiety relief and stress management, a study conducted in 2005 indicated a decline in anxiety, stress, and improved mood following inhalation of the scent of lavender oil [12]. Moreover, a 2012 study on postpartum women indicated that aromatherapy using lavender oil for 15 min twice a week for four weeks lowered anxiety levels and caused a decline in depression levels [13]. In 2015, an improvement in sleep, energy, and vibrancy was noted in students who suffered from sleep deprivation and inhaled the scent of lavender oil before bedtime [14]. Another research group also observed this sleep-promoting effect in 2015 [15]. In 2018, geriatric populations with enhanced duration and sleep quality followed aromatherapy involving lavender oil [16]. Taken together, these studies further confirm the exertion of the oil’s anxiolytic effect through interactions with the GABAergic system.

- Lemongrass oil

The major constituents of the oil are citral (a mixture of niral and geranial) and β-myrcene [2,19]. An animal model study conducted in 2011 determined that the anxiolytic activity of lemongrass oil at a dose of 10 mg/kg (p.o) possibly occurs through interaction with the GABA receptor–benzodiazepine complex as the effect of lemongrass oil was inhibited by flumazenil, a competitive antagonist of benzodiazepines [20,21]. Moreover, in a study conducted in 2015, the aroma of lemongrass (from three to six drops) brought about a significant decline in stress and anxiety in subjects [22]. Similar to lavender oil, lemongrass oil is believed to exert its effect through interactions with the GABAergic system [2].

### 3.3. Role in Depression Management

According to the NIH, depression is a prevalent mental disorder and can occur due to genetic, biological, environmental, and psychological factors or a combination of these factors. Treatments include a form of psychotherapy such as electroconvulsive therapy (ECT) and antidepressants [96]. The complications associated with depression include weight gain, social isolation, self-mutilation, pain, alcohol and drug abuse [97]. Essential oils that can use for mood improvement and symptom alleviation of depression include:

- Ylang ylang oil

**Figure 5:**
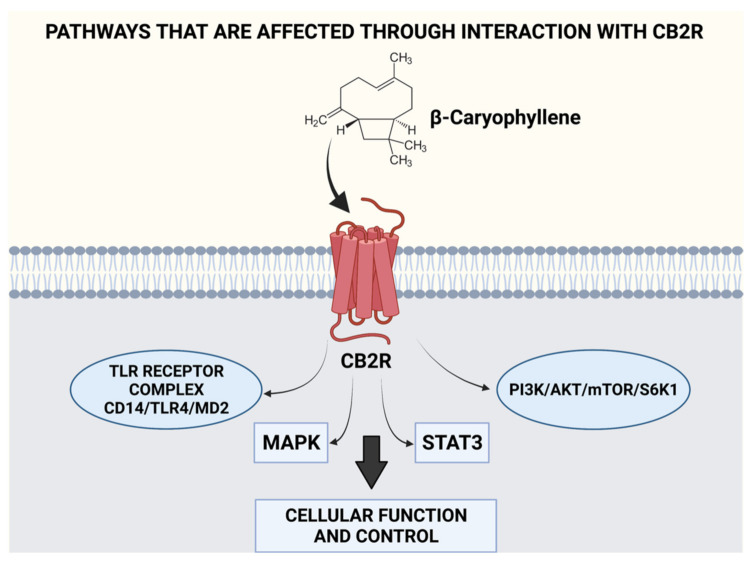
Interaction of EOs with the ECS leads to the modulation of several pathways. Created using Biorender.com. (Last accessed on 14 December 2021).

This essential oil consists of approximately 150 identified compounds. However, the mood adjustment and relaxation effect provided have been attributed to β-caryophyllene, benzyl benzoate, linalool, and benzyl alcohol in the oil [23,24]. In 2013, the impact of ylang-ylang EO was studied on 15 healthy men wherein three drops of the oil were added to a warm water lamp maintained at 90 °C in an enclosed space. After 60 min of exposure, the subjects’ heart rate and blood pressure levels decreased along with a simultaneous decline in the activation of the autonomic nervous system (ANS) [25]. In 2018, a study showed that inhalation of ylang ylang essential oil by anxious mice caused a decline in CREB and Fos-c in the hippocampus, decreased plasma corticosterone, and altered blood serotonin metabolism [26]. In 2016, the mechanism behind the anxiolytic and mood adjusting effect was identified to occur through effects on the serotoninergic (5-HT) and dopaminergic pathways (DA) and was attributed to the presence of benzyl benzoate in the oil [24]. Moreover, its major constituent, β-caryophyllene, is also associated with anti-inflammatory, anticancer, neuroprotective, antioxidant, and mood-adjusting effects [98,99,100]. Studies have indicated that the mood adjusting effects of ylang ylang oil occur due to the direct binding of β-caryophyllene to CB2R receptors located on several organs, which cause the modulation of ECS activity, thereby controlling responses (both cognitive and emotional) to stressors through ECS interactions [28]. The ability of β-caryophyllene to modulate various pathways and possess multiple benefits through the CB2R receptor has been illustrated in Figure 5. β-caryophyllene has been found to hinder metastasis, cause a reduction in oncogene and protein expression of cancer cells while upregulating genes and proteins that destroy cancer cells through the modulation of pathways such as MAPK, PI3K, AKT, mTOR, S6K1, and STAT3. Therefore, its use is suggested for kidney, lung, oral, liver, lymphoma, and neuroblastoma cancers due to its chemo preventive activity [93]. β-caryophyllene when administered orally, has been found to inhibit CD14/TLR4/MD2 toll-like receptor complex that is responsible for the production of pro-inflammatory cytokines, such as IL-1β, IL-8, IL-6, and TNF-α, while also causing the synergy of μ-opioid receptor pathways [101,102]. Moreover, it has also been attributed to modulating pain signaling pathways in a synergistic manner with other analgesic substances [103]. Figure 5 illustrates the various pathways affected through interaction with the ECS.

- Cinnamon oil

The oil consists of 15 identified compounds, and the principal component (65–85%) is trans-cinnamaldehyde which is responsible for the mood adjustment effect of the oil. Antidepressant effects in albino male mice were observed following intraperitoneal (i.p) injection (3 in 24 h/1 per day for 14 days) using doses of 0.5, 1, and 2 mg/kg [29]. The mechanism of this effect remains unknown. However, in 2016, another group of researchers suggested the downregulation of nitric oxide synthase, cyclo-oxygenase 2 (COX-2), and TNF-α, and suppressing neuroinflammation and NF-κB and p53 in activated B-cells was responsible for the antidepressant effect observed [30]. In contrast, Iwasaki et al. (2008) showed that intravenous (IV) administration of TCAs caused an upregulation of adrenaline secretion through adrenal sympathetic nerves along with the activation of sensory nerves that express thermosensitive transient receptor potential channels A1, thus being beneficial in monoamine-associated depressive disorders where a decline in adrenaline level is observed [31]. Therefore, further research to determine the mechanism by which cinnamon oil exerts its antidepressant effect is needed.

### 3.4. Role in Memory Retention, Neuroprotection, and Alzheimer’s Disease Management

**Figure 6:**
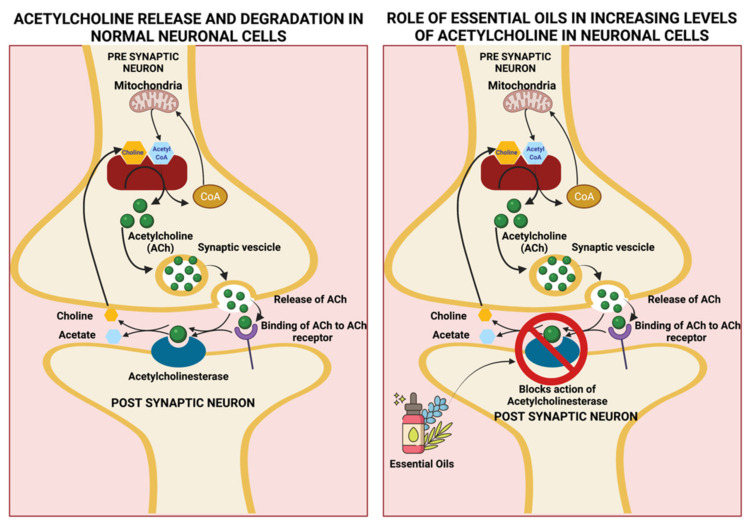
Ability of EOs to inhibit acetylcholinesterase (AChE), thereby increasing levels and duration of acetylcholine in the brain and assisting with memory retention. Created using Biorender.com. (Last accessed on 14 December 2021).

Disruption of daily life due to memory loss could be an early sign of dementia or Alzheimer’s disease. Patients diagnosed with Alzheimer’s find it difficult to perform daily tasks and lose track of dates, seasons, important events, and time. Due to progressive memory loss that occurs over time, patients find it difficult to remember. Causes of Alzheimer’s disease are poorly understood. The disease is associated with the presence of amyloid plaques, neurofibrillary tangles, and loss of neural connections in the brain [104,105]. Several EOs have been found to be beneficial for symptom reduction and disease treatment of Alzheimer’s disease through various mechanisms such as acetylcholinesterase inhibition illustrated in Figure 6, nicotinic/GABA_A_ receptor interactions, etc. These EOs and the mechanisms by which they cause Alzheimer’s disease symptom alleviation are discussed below:

- Eucalyptus oil

The leaves’ major constituents responsible for its CNS activities are 1,8-cineole (Eucalyptol) and α-pinene [33]. Eucalyptol is a monoterpenoid, is the major component (90%) of eucalyptus oil, and is well known to provide an anti-inflammatory, mucolytic, and spasmolytic effect on the respiratory tract, thus aiding to relieve inflammatory diseases such as asthma and chronic obstructive pulmonary disease (COPD) [34]. Another component of eucalyptus oil is α-pinene, which exists as a racemic mixture. Besides exerting anti-inflammatory [35] and antimicrobial effects [36], it exerts an inhibitory effect on acetylcholinesterase (AChEI), the enzyme responsible for the breakdown of the neurotransmitter acetylcholine into choline and acetate [37], the results of which leads to enhanced levels and duration of acetylcholine in the CNS, thereby aiding memory as shown in Figure 6 [35]. The acetylcholinesterase inhibitory effect and mechanism of action of α-pinene is therefore beneficial for the prevention and progression of neurodegenerative disease such as Alzheimer’s disease, which is associated with a decline in levels of acetylcholine due to cholinergic neuron deterioration, which results in memory loss-an important characteristic of the neurodegenerative disease [1].

- Peppermint oil

The oil contains 26 identified volatile compounds, most of which are oxygenated monoterpenes such as menthol and iso-menthone [2,39]. Others include limonene, cineole, menthofuran, menthyl acetate, isoeugenol, pulegone, and carvone [40]. Umezu et al., in 2012, determined the CNS stimulant activity of peppermint oil using a discrete shuttle-type conditioned avoidance task in mice [41]. Similarly, a study conducted by Kennedy et al. in 2018 indicated that the improvement in mood effects and cognitive tasks and decline in mental fatigue in individuals administered with 100 µL of peppermint oil occur through nicotinic/GABA_A_ receptor binding and acetylcholinesterase inhibition (Figure 6) [42]. It also possesses an antioxidant effect, increases glutathione, and prevents oxidative stress. When administered at a lower dose (100 mg/kg s.c), improvement in spatial working memory was observed in mice, while at a higher dose, a decline of malondialdehyde (a lipid peroxidation product) occurs in aged and ß-amyloid treated mice, thus improving cognitive function. When administered in mice for ten days, an improvement in spatial learning and memory along with the reversal of amnesia upon treatment with ß-amyloid was observed, proving its benefits in preventing Alzheimer’s disease and memory [43].

- Rosemary oil

The oil contains more than 16 identified compounds, majorly being camphor, cineole, α-pinene, camphene, and α-terpineol [2,46]. Besides possessing anxiolytic properties, rosemary oil also aids in memory, mood, and cognitive functions. In 2017, the inhalation of rosemary oil by mice increased dopamine levels while decreasing immobility time and serum corticosterone levels. The mechanism behind the effects occurred through intracellular modulation of acetylcholine, choline, and Gap43 gene expression levels. Moreover, rosemary oil was found to affect the stress response system through the nerve growth factor (NGF) pathway and the hypothalamus–pituitary–adrenal axis, thus bringing about dopamine activation (DAergic system activation) secretion. The authors attributed this effect to α-pinene, a known anxiolytic [47]. Rosemary oil also offers antioxidant-mediated neuronal protection against brain inflammation and ß-amyloid plaques observed in Alzheimer’s disease [48].

- Sage oil

Sage oil contains camphor, α-thujone, 1–8, cineole, viridiflorol, β-thujone, β-caryophyllene, and 49 other constituents [2,51]. A study conducted in 2014 showed that sage oil could modulate retrospective memory, attention, and mood [52] by acting as an acetylcholinesterase inhibitor. Sage oil also acts as a powerful antioxidant, enhances antioxidant defense systems, and prevents lipid oxidation, beneficial for induced acquisition and memory deficits observed in diabetic patients [53]. Moreover, it has shown beneficial effects on patients with mild to moderate Alzheimer’s disease. After four months of usage of sage oil (fixed dosage 60 drops/day), patients showed improved cognitive function [54].

- Sandalwood oil

Sandalwood oil consists mainly of tricyclic α-santalol and β-santalol [56]. A study conducted in 2020 by Younis et al. showed that sandalwood essential oil improved neurological deficits decreased oxidative stress and inflammatory cascade in mice subjected to middle cerebral artery occlusion surgery (MCAO) [57]. The methanolic extracts of sandalwood administered to albino mice showed acetylcholinesterase inhibitory effect along with α, α-diphenyl-β-picrylhydrazyl (DPPH) superoxide radical free scavenging activities, thus proving beneficial to prevent the progression of dementia and loss of memory in Alzheimer’s patients [58,59]. A 2016 study on 32 humans showed a reduction in blood pressure and salivary cortisol levels, indicating its benefits in stress reduction as well [60].

## 4. Essential-Oil-Based Nanomedicines/Pharmacotherapy

The major requirement of any therapeutic targeting the CNS is to cross the blood–brain barrier (BBB), which protects the brain from circulating toxins and pathogens and controls the transport of serum factors and vital nutrients [106]. Lipophilic drug molecules of molecular weight between 400 and 600 Daltons (Da) have facile entry through the BBB through passive diffusion or solubilization in the lipid bilayer of the endothelial cell membrane. This, unfortunately, allows for the administration of only a handful of drug moieties [107].

The advent of nanotechnology, which focuses on the development of particles whose sizes are less than 100 nanometers, is promising and can provide solutions for targeted drug delivery to the CNS and the crossing of the BBB. Researchers successfully coupled therapeutic agents along with polymer NP’s, liposomes, and micelles in the past decade. However, owing to their inability to provide for neuronal repair and regeneration as well as the failure to completely penetrate the BBB, novel advanced nano delivery systems such as dendrimers, nano gels, nanosuspensions, nanotubes, etc., have been developed, which utilize functionalized nanomaterial that allows for enhanced drug delivery via endocytosis and transcytosis [108]. These novel technologies and their therapeutic applications are described below:

- Dendrimers

These comprise a 3D structure containing an initial core, multiple internal layers, repetitive units, and several active terminal surface groups [109]. The increase in dendrimer branches is dependent on the intensity of dendrimer generation [110]. As a result, dendrimers offer properties such as low dispersion and high performance [111]. Due to the presence of many surface groups and a hydrophobic core, a large dose/concentration of drugs/imaging agents can be loaded onto dendrimers [112]. The stages pertinent to the coupling of ligands to dendrimers for therapy are as follows:

Stage 1: Modification of dendrimers with distance or surface linkages to enhance biocompatibility and pharmacokinetic parameters pertaining to drug release [113].

Stage 2: Drug/ligands coupled to the dendrimer undergo structural modifications to improve BBB penetration or tumor targeting and drug delivery [114,115].

Stage 3: Complex biological junctions are formed by modified dendrimers used in drug or gene therapy [116,117].

Stage 4: Covalent coupling of imaging agents to dendrimers to allow for imaging and in vivo diagnostics [114,115].

Sharma et al. developed, through click chemistry, facile neuroinflammation targeting PEG-based dendrimer (PEGOL-60) and showcased its efficient penetration into the brain and glial targeting through achieving at low generation, a high hydroxyl surface density. Systemic administration of PEGOL-60 targeted activated microglia and macrophages at the site of injury in various animal models of cerebral palsy, glioblastoma, and age-related macular degeneration indicating facile drug delivery and penetration through the brain [118].

- Nanogels

These constitute a network of polymers in the nanoscale, which form ionic and non-ionic chains such as polyethylene amine (PEA) and polyethylene glycol (PEG) [119]. They possess a 40–60% drug loading capability, which is not possible with other nanocarrier systems. The utilization of hydrogel-based nanoparticles has gained significance owing to the simultaneous possession of both hydrogel and nanoparticle characteristics [120].

Surface modification of the nanogels with transferrin and insulin renders an enhanced distribution through the BBB [121]. In vivo studies performed indicate that oligonucleotide delivery to the brain is enhanced along with the decreased absorption by the spleen and liver when nanogels are used [122]. As a result, nanogels have proven to be very promising candidates in the delivery of drugs to the CNS [120]. Azadi et al. developed an anticancer drug methotrexate-based nanogel formulation. They observed that the following injection into the bloodstream and subsequent binding to apolipoproteins, nanogel molecules were attached to the endothelial cells of brain capillaries which further diffused into the endothelial cells through endocytosis [123]. Similar results were obtained by Gulyaev et al. when studying the transportation of doxorubicin using polysorbate 80 coated nanoparticles [124].

- Carbon nanotubes (CNTs)

These comprise carbon-based cylindrical nanostructures and possess multiple layers of carbon; thus, they are characterized as either single-wall or multiwall CNTs [125]. Over the years, unmodified and modified CNTs have been evaluated for their therapeutic efficiency [126,127]. The permeability of amino-functioned single-walled CNTs using a scanning electron microscope in an animal model was studied by Kafa et al. and showed an enhanced accumulation in the brain tissue and increased astrocyte uptake. A notable observation was that of decreased permeability to the brain with temperature elevation indicating a drug delivery mechanism that is energy-dependent [128].

Aziz et al., in 2019, via spontaneous emulsification, developed an optimized nano emulsion system containing eucalyptus micelles using surfactants such as Tween 40, 60, and 80 and concentrations between 3 and 18% wt. They were further characterized for their thermodynamic stability, particle size, pH, morphology, and viscosity. The studies revealed that utilization of Tween 40 at surfactant concentration of 9 wt. % provided the best results. In vivo transdermal application of these micellar nanoparticles (100 mg/kg) on rats’ fore and hind limbs provided central and peripheral analgesic effects noted through a prolonged pain response at 40.75 s. Versus the application of pure Eucalyptus EO (500 mg/kg), which provided a response time of 34.5 s when rats were placed on a 55 °C hot plate (heat stimulus) [129].

Scuteri et al. developed a cream-based formulation using a solid lipid nanoparticulate delivery system with bergamot EO. Bergamot is known to possess strong antinociceptive and anti-allodynic properties. The developed nanoparticle formulation provides stability of the titrated bergamot components, was studied for its in vivo analgesic effects, is devoid of smell, and is currently under patent consideration for use in agitation control in patients diagnosed with severe dementia [130]. Taken together, the coupling of essential oils with nanocarriers is promising and could provide breakthrough solutions for the treatment of neurological disorders.

## 5. EO Therapy: Challenges and Research Gaps

- Lack of sufficient information pertaining to utility and dosage

Although EOs offer multiple benefits, modern approaches for disease treatment and symptomatic relief revolve around the use of synthetic chemicals associated with adverse effects such as nausea, drowsiness, stomach irritation, etc., to name a few [131]. As a result, some patients and clinicians then turn to use natural products whose benefits are well-known, such as the antibacterial action of turmeric, the analgesic effect of clove oil, etc., [132]. The use of natural products for therapy is a form of alternative medicine that has expanded over the years. However, even though some EOs have been traditionally used for the treatment of various ailments and can target various organs of the body inclusive of the CNS, clinician, and patient utilization so far, is predominantly limited to external applications of oils, aromatherapy, and spa treatment for muscular pain or stress relief [132]. This then renders the pharmacological action of EOs and their applicability for disease treatment an incompletely explored domain. For instance, a recent paper published in 2021 indicated that some constituents of EOs had the ability to cause maternal toxicity, abortions, teratogenicity, and embryo-fetotoxicity [133]. Therefore, the use of EOs in various disease models and patient types (gender differences, geriatrics, pediatrics, disease models, pregnancy, etc.) needs further development and investigation to be considered as a bona fide alternative for disease treatment.

- Patient Acceptability of EOs

A study conducted by Conlon et al. pertaining to the acceptability of EOs in pediatrics and other practices revealed that some patients had negative comments on the use of EOs for therapy, such as allergic reactions (e.g., sneezing, tearing of eyes, as well as difficulty remembering the indications and usage of each oil). Moreover, since some oils are unpleasant in aroma, and patients revealed that they preferred using a combination of EOs with a pleasant fragrance as compared to a single oil with an unpleasant aroma. The study thus highlighted the need for accommodation of individual preferences to improve patient acceptability of EOs in therapeutics [134].

With respect to the use of EO-based nanoparticles for pharmacotherapy, safety is an important concern. Although a majority of EOs has been deemed as GRAS (generally regarded as safe), Refs. [135,136] controversial data were obtained that need further investigation. Lalko and Api tested skin irritancy of EOs and their isolated constituents as topical formulations on 8–12-week CBA/Ca female mice using five concentrations ranging from 2.5 to 50% *w/v* in 1:3 ethanol: diethyl phthalate. They observed a dose-dependent sensitization upon exposure and contact with the oils and their constituents, particularly oils that contained citral, eugenol, and geraniol. However, unexpectedly, even though basil oil majorly consists of linalool and eugenol, it produced a higher EC3 (estimated concentration) value. Unexpected results were also obtained for citronella oil and geranium oil that produced a low level of sensitization considering their high geraniol content [137]. Similar results were obtained by Opdyke in 1976. Opdyke reported sensitization was brought about by cinnamic aldehyde, phenylacetaldehyde, and citral. However, again unexpectedly, EOs that contained significant concentrations of these constituents did not induce sensitization, indicating that other component(s) were responsible for sensitization. To test this hypothesis, mixtures containing aldehydes, terpenes, and alcohols were tested. These mixtures showed no induction of sensitivity, while that of individual aldehydes produced sensitivity. This concept of masking the sensitizing reaction was termed as ‘quenching’ and was incorporated into risk management strategies for contact allergy [138]. Over the years, the phenomenon of quenching was tested. However, conflicting results were by several research groups. For instance, carvone, a fragrant ingredient and a well-known sensitizer (mechanism for antigen formation is described) [139], was found to be inhibited by structural and non-structural analogs in guinea pigs [140,141]. On the other hand, studies on guinea pigs revealed no quenching of cinnamic aldehyde and citral [142]. As a result, due to conflicting data and the lack of conclusive evidence to explain the mechanism of quenching, it has been replaced as a basis for risk management of contact allergy of certain fragrances. For example, the standard pertaining to cinnamic aldehyde according to the International Fragrance Association (IFRA) was revised to limit usage based on its NOEL (maximum tested no observed effect level) [137].

It is also worth noting that the contact sensitization of EOs containing known sensitizers such as limonene and linalool was found to be dependent on the oxidation state of these components. Upon auto-oxidation, both components were found to form products that cause that have the potential to cause allergies [137]. Therefore, further studies to develop analytical approaches for understanding oxidation processes and prevention strategies are warranted and are a valuable future objective for the development of safer therapeutics.

- Sustainability

The recent decade has witnessed several efforts to lower environmental pollution through the synthesis of compounds using ‘green chemistry’ [135]. As EOs are naturally occurring and are thus ‘greener’ as compared to organic solvents, their utilization as a substitute of organic solvents used in the preparation of nanocapsules [143] and metallic nanoparticle [144] synthesis has been studied. Efforts have also been made for the development of greener EO extraction methods with minimum or lack of solvent and energy utilization to obtain high-grade and high-quality EOs [145]. Because EOs show little to no toxicity and have high potential as CNS-based therapeutics, the development and utilization of high throughput, high yielding, greener techniques for EO-based CNS therapeutics synthesis and development seems promising, especially for pharmaceuticals for aiding in balancing investments involved in the development and implementation of sustainable alternatives [146,147].

## 6. Discussion

EOs are complex, volatile mixtures that comprise several low molecular weight constituents such as monoterpenes and sesquiterpenes that are biologically active. While sometimes the major active constituent is responsible for the activity of EO, several EOs possess many other constituents that provide a wide variety of benefits. Moreover, in some instances, an enhancement in biological activity (synergy) is observed in EOs comprising of multiple constituents as compared to when these constituents are isolated. These EOs warrant attention because their significance and utilization as healing systems have been well-established throughout history.

The aforementioned studies suggest that EOs act directly and indirectly on the CNS through targeting various receptors and pathways. Thus, they can be used for the treatment and symptomatic reduction of multiple disease states. For instance, direct-acting EOs containing β-caryophyllene have been found to modulate immune function and inflammatory responses through the regulation of immune cells expressing CB2R such as dendritic cells, macrophages, eosinophils, etc., which is further relevant and plays a critical role in alleviating the dysfunctionalities characteristic of inflammatory conditions such as cancer and neurodegenerative diseases [148,149]. EOs have also been found to assist in the co-operative working CB2R and CB1R, which further aids in alleviating symptoms associated with neurological diseases [150]. An enhanced MAGL enzymatic activity coupled with upregulation of FAAH has been observed through a post-mortem of patients’ brains with Alzheimer’s disease [151,152]. EOs’ ability to cause neuroprotective action, activation of CB2R through direct binding of β-caryophyllene coupled with their ability to cause downregulation of FAAH and MAGL activity support their use to modulate both the innate immune responses of the brain and the disease progression in Alzheimer’s patients.

Positive benefits have also been observed through AChE inhibition, interactions with the DAergic, nicotinic/GABA_A_, 5-HT systems, etc. Therefore, harnessing the multiple benefits of EOs through the development of oil blends and targeted therapeutics to modulate CNS activity, directly and indirectly, could aid in the rapid establishment of homeostasis, symptom reduction, disease progression prevention, and cure of CNS-based disorders [153,154,155].

With respect to the routes of administration, preclinical and clinical data obtained suggest that following olfactory and oral administration of EOs, activation or inhibition of certain components or areas of the brain is related to the olfactory receptors, which is a very fascinating fact since these receptors are expressed not only on the outside the nasal cavity but also in various organs such as the GI tract, lungs, kidney, and heart [100,156].

Although the challenges pertaining to the development of novel EO-based therapeutics require consideration, due to the growing number of patients affected by neurological diseases, researchers should work towards ensuring significant advancements in the field of nanotechnology, which can finally culminate into the development of effective EO-based targeted therapeutics, thus saving millions of lives, and improving the quality of life of patients affected with these unfortunate diseases.

## 7. Conclusions

The constituents of essential oils synergistically exert their effects to produce a diverse range of pharmacological and physiological effects. The mechanism of action of these constituents and the various systems affected has been identified through the research conducted on animal models and humans over the years. Substantial evidence through preclinical and clinical data has been obtained proving the influence of essential oils on the sympathetic nervous system and neurotransmitter systems such as DAergic, GABAergic, and serotoninergic systems. However, as described, the mechanism of action of some essential oils on the CNS is yet to be elucidated.

The ability of essential oils to produce a wide range of therapeutic effects through action on various neural pathways and their low potential to cause adverse reactions makes them ideal candidates for therapy of CNS-based disorders. The development of proper models for biological activity analysis coupled with further research on the binding, synergy of constituents, and stability of the complex formed between CNS receptors and essential oils would thus assist in paving the way for the successful development of EO-based medications and EO-based targeted therapy nanomedicines.

## Figures and Tables

**Table 1:** Summary of the active constituents, extraction technique, effective doses, biological activity, and uses of EOs targeting the CNS.

Plant/Source	Active Constituents (>20%)	Extraction Method	Effective Dose/Preparation Technique	Biological Activity	Uses	References
Syzygium *aromaticum*	Eugenol (76.8%)	Water or steam distillation of the buds, stem, and leaves of clove tree	50/100/200 mg/kg of aqueous extract/ethanolic extract of clove oil. Aqueous extract showed better results	GABA_A_ receptor agonist	Analgesic	[4,5,6]
Boswellia *sacra*, Boswellia *frereana*	α-Pinene (2–64.7%), α-thujene (0.3–52.4%), myrcene (1.1–22.4%), limonene (1.3–20.4%)	Hardened aromatic gum resins obtained from the tree	50 μL in a 1:1000 dilution with jojoba oil on the nape of neck for 5 h with hourly intervals	Undetermined, believed to occur due to the synergistic effect of constituents	Anxiolytic and stress relief	[2,7,8,9]
Lavandula *angustifolia*	Linalyl acetate (7.4–44.2%), linalool 11.4–46.7%)	Steam distillation of flowers	80 mg of standardized product (Silexan available in Germany) containing 36.8% of linalool and 34.2% linalyl acetate 160 mg/day for 8 weeks	GABAergic system interaction Antagonist of NK-1 receptor inhibiting release of substance P reduces peripheral and central nerve excitability Inhibition of voltage-gated calcium channels, reduction of 5-HT1A receptor activity, and increased parasympathetic tone	Anxiolytic, stress relief, mood enhancement, analgesic, and pain relief	[10,11,12,13,14,15,16,17,18]
Cymbopogon *citratus*	Citral (26.1%), neral (31.5%)	Steam distillation of fresh or partially dried grass	1–10 mg/kg per day for 14 days	GABAergic system interaction	Anxiolytic, stress relief, and mood enhancement	[19,20,21,22]
Cananga *odorata*	β-Caryophyllene (26.8%)	Stem distillation of the flowers	1% v/v of ylang ylang oil for 10 min. 25/50/100 mg/kg of β-Caryophyllene was injected intraperitoneally	Activation of ANS and has effects on the 5-HT and DAergic system Direct binding onto CB2R receptor	Mood adjustment, relaxation, and antidepressant activity	[23,24,25,26,27,28]
Cinnamomum *verum*	Trans-cinnamaldehyde (71.50%)	Brown bark	0.5–2 mg/kg body weight three times a day or once daily for 14 days	Undetermined	Mood elevation and antidepressant action	[29,30,31,32]
Eucalyptus *globulus*	1,8-cineole (49.07–83.59%), α-pinene (1.27–26.35%)	Steam distillation of the leaves	3% v/v dissolved in almond oil, 30 min daily for 3 days	Acetylcholinesterase inhibition	Anti-inflammatory, improves memory, and improves symptoms of Alzheimer’s disease	[33,34,35,36,37,38]
Mentha *piperita*	Menthol (40.7%), iso-menthone (23.4%)	Stem distillation of the leaves	4 drops of oil in a diffuser pad followed by 5 min of inhalation 2500 µL capsules containing 50–100 µL of peppermint oil in vegetable oil	Binds to the nicotinic/GABA_A_ receptor and inhibits acetylcholinesterase	CNS stimulation, antioxidant, and memory retention	[39,40,41,42,43,44,45]
Rosmarinus *officinalis*	p-Cymene (44.02%), linalool (20.5%) 1,8-cineole (26.54%), α-pinene (20.14%),	Hydro distillation of the aerial parts	4 drops of oil in a diffuser pad followed by 5 min of inhalation	Improves DA activation and secretion	Anxiolytic, improves mood and cognitive function	[46,47,48,49,50]
Salvia *sclarea*	Camphor (12.8–21.4%), α-thujone (17.2–27.4%), 1–8, cineole (11.9–26.9%),	Hydro distillation of the aerial parts	5 drops of EO in 5 mL of water along with an aroma stone	Acetylcholinesterase inhibition	Improves memory, mood, attention and is beneficial for mild to moderate severity of Alzheimer’s disease	[51,52,53,54,55]
Santalum *paniculatum*	α-santalol (34.5–40.4%) and β-santalol (16–24.10%)	Steam distillation of the heartwood and roots	1 g/kg body weight of sandalwood oil in 5% Tween 80 in saline for a week	Acetylcholinesterase inhibition	Improves memory, prevents dementia, beneficial in Alzheimer’s disease	[56,57,58,59,60,61,62]

**Table 2:** Summary of the preclinical and clinical data pertaining to the CNS activity of EOs.

Plant/Source	Essential Oil	Test Subjects/Animal Model	Route of Administration Tested	Effective Dose/Preparation Technique	Experimental Outcome	Purpose of Use	References
Syzygium *aromaticum*	Clove oil	90 BALB/male mice (27–32 g)	Intraperitoneal injection	50,100, and 200 mg/kg of aqueous/ethanolic extract of clove in a final volume of 10 mL/kg	Maximal percent effect (MPE) of animals that were tested on hot plate and treated with oil was higher than that of the control group	Analgesic	[4]
Boswellia *sacra,* Boswellia *frereana*	Frankincense oil	Adult male Sprague Dawley sleep-deprived rats	Topical application	50 μL in a 1:1000 dilution with jojoba oil on the nape of neck for 5 h with hourly intervals	Corticosterone and glutathione levels declined, wakefulness time increased, and non-rapid eye movement time declined	Antidepressant, mood elevation, anxiolytic, and stress relief	[8]
Lavandula *angustifolia*	Lavender oil	200 pregnant women undergoing cesarean section	Olfactory administration	2 drops (1% cc) of 2% lavender essence applied with a cotton swab to oxygen face mask, which was used for 3 min, repeated thrice over different time periods	Mean Visual Analogue Scale (VAS) decreased significantly, indicating amelioration of pain	Analgesic	[63]
Cymbopogon *citratus*	Lemongrass oil	30-day old adult swiss male mice	Oral administration	Doses of 1, 5, and 10 mg/kg provided as well as repeated dosing for 14 days	Anxiolytic effects observed through results obtained in light/dark box test	Anxiolytic	[20]
Cananga *odorata*	Ylang ylang oil	29 male participants	Olfactory administration	Participants placed in a closed room for 60 min that was previously fragranced with ylang ylang oil for 20 min.	Decline in systolic and diastolic BP and reduction in heart rate	Sedative effect and mood adjustment	[25]
		Male and female mice weighing 25–30 g and 22–25 g, respectively	Olfactory administration	Stainless steel square inhalation apparatus (65 × 65 × 45 cm) with controllable heater to heat oil/water emulsion containing ylang ylang oil (1% *v*/*v*) and benzyl benzoate (2% *v*/*v*). Mice placed in chamber for 10 min	Male mice experienced more changes in concentration of neurotransmitters than female mice. Decline in DA in striatum and 5-HT concentration in hippocampus and decreased ratio of 5-HIAA/5-HT	Anxiolytic effect on male mice	[24]
Cinnamomum *verum*	Cinnamon oil	Male albino mice	Intraperitoneal injection	0.5–2 mg/kg body weight three times a day or once daily for 14 days	Decreased immobility time in forced swim test (FST) and tail suspension test (TST) Mice treated with 2 mg/kg spent a longer time and showed more entries into the open arms of elevated plus-maze (EPM)	Antidepressant and anxiolytic	[29]
Eucalyptus *globulus*	Eucalyptus oil	28 individuals with osteoarthritis that underwent total knee replacement surgery	Olfactory administration	3% *v/v* was dissolved in almond oil, placed on 4 × 2 gauze pad, and inhaled for 30 min for 3 consecutive days	VAS scores after aromatherapy decreased. Heart rate increased to 0.3+/− 0.6 beats/min on day 1 and decreased to 1.7+/−1.7 beats/min and 0.6+/−1.0 beats/min on days 2 and 3, respectively	Analgesic, lowering BP, stress relief, and anxiolytic	[38]
Mentha *piperita*	Peppermint oil	144 healthy individuals 24 participants (9 male/15 female) (mean age 25.2 years)	Olfactory administration Oral administration	4 drops of oil in a diffuser pad followed by 5 min of inhalation 2500 µL capsules containing 50–100 µL of peppermint oil in vegetable oil	Enhanced alertness and memory 100 µL dose caused an improvement in rapid visual information processing task (RVIP) performance at 1 h and 3 h post-dose. Both doses decreased fatigue	Memory booster, modulated performance on cognitive tasks, and decreased mental fatigue	[42,45]
	Rosemary oil	20 healthy individuals	Olfactory administration	Inhalation of 10% *v*/*v* of the oil for 20 min using an oxygen pump attached to a respiratory mask whose airflow rate is 2 L/min	Decreased both powers of alpha1 and alpha2 waves	CNS stimulant	[64]
Rosmarinus *officinalis*		140 healthy individuals	Olfactory administration	4 drops of oil in a diffuser pad followed by 5 min of inhalation	Mood elevation increased blood pressure, heart rate Improved mood and enhanced quality of memory	Memory enhancer	[62]
Salvia *sclarea*	Sage oil	45 healthy individuals 135 healthy individuals	Olfactory administration Olfactory administration	5 drops of EO in 5 mL of water along with an aroma stone 5 drops of EO in 5 mL of water along with an aroma stone	Memory enhancement Improved and enhanced memory and secondary memory	Memory enhancement Memory enhancement	[52,65]
Santalum *paniculatum*	Sandalwood oil	D-galactose mediate oxidative stress-induced Swiss male albino mice (20–30 g)	Intraperitoneal administration	1 g/kg body weight of sandalwood oil in 5% Tween 80 in saline for a week	Oxidative stress status ameliorated in group-administered sandalwood oil. Recovery of GSH, NO levels, catalase, and lipid peroxidation status in liver. Reduction in serum bilirubin, SGOT and SGPT.	Antioxidant	[59]

## Data Availability

Not applicable.

## Abbreviations

EO: Essential oil, CNS: Central nervous system, CBT: Cognitive behavioral therapy, SSRIs: Selective serotonin reuptake inhibitors, TCAs: Tricyclic antidepressants, NMDA: N-methyl-D-aspartate, WHO: World Health Organization, GAD: Generalized anxiety disorder, PD: Panic disorder, OCD: Obsessive-compulsive disorder, PTSD: Post-traumatic stress disorder, PDD: Pervasive developmental disorder, GABA: Gamma-aminobutyric acid, NIH: National Institute of Health, ECT: Electroconvulsive therapy, ANS: Autonomic nervous system, COPD: Chronic obstructive pulmonary disease, AChEIs: Acetylcholine esterase inhibitors, c-AMP: 3′, 5′-cyclic adenosine monophosphate, CREB: c-AMP response element-binding protein, 5-HT: Serotonin, DA: Dopamine, COX-2:Cyclo-oxygenase 2, TNF-α: Tumor necrosis factor-alpha, NF-κB: Nuclear factor kappa light chain enhancer of activated B cells, Gap43: Growth associated protein, NGF: Nerve growth factor, MCAO: Middle cerebral artery occlusion, DPPH: α, α-diphenyl-β-picrylhydrazyl hydrate, TRP: Transient receptor potential, SWS: slow-wave sleep, ACC: anterior cingulate cortex, PFC: prefrontal cortex, PAG: periaqueductal gray, BP: Blood pressure, 5-HIAA: 5-hydroxyindoleacetic acid, MPE: Maximal percent effect, SGOT: serum glutamic oxaloacetic transaminase, SGPT: serum glutamic pyruvic transaminase, BBB: Blood–brain barrier, Da: Daltons, CNT’s: Carbon nano tubes, ECS: Endocannabinoid system, MAGL: Monoacylglycerol lipase, AEA: Anandamide, AG: 2-Arachidonoylglycerol, CD14: Cluster of differentiation 14, TLR4: Toll-like receptor 4, MD2: Myeloid differentiation factor 2, IL-1β-Interlukin 1-beta, IL-interlukin, GRAS: Generally regarded as safe, EC3: Estimated concentration, IFRA: International Fragrance Association, NOEL: No-observed-effect level.

## References

[1] Ayaz M., Sadiq A., Junaid M., Ullah F., Subhan F., Ahmed J. (2017). Neuroprotective and Anti-Aging Potentials of Essential Oils from Aromatic and Medicinal Plants. Front. Aging Neurosci..

[2] Lizarraga-Valderrama L.R. (2021). Effects of essential oils on central nervous system: Focus on mental health. Phytother. Res..

[3] ChStratakos A., Koidis A., Preedy V.R. (2016). Methods for Extracting Essential Oils. Essential Oils in Food Preservation, Flavor and Safety.

[4] Asl M.K., Nazariborun A., Hosseini M. (2013). Analgesic effect of the aqueous and ethanolic extracts of clove. Avicenna J. Phytomed..

[5] Xu H., Delling M., Jun J.C., Clapham D.E. (2006). Oregano, thyme and clove-derived flavors and skin sensitizers activate specific TRP channels. Nat. Neurosci..

[6] Jirovetz L., Buchbauer G., Stoilova I., Stoyanova A., Krastanov A., Schmidt E. (2006). Chemical composition and antioxidant properties of clove leaf essential oil. J. Agric. Food Chem..

[7] Mertens M., Buettner A., Kirchhoff E. (2009). The volatile constituents of frankincense—A review. Flavour Fragr. J..

[8] Okano S., Honda Y., Kodama T., Kimura M. (2019). The Effects of Frankincense Essential Oil on Stress in Rats. J. Oleo Sci..

[9] Vuuren S.F.V., Kamatou G.P.P., Viljoen A.M. (2010). Volatile composition and antimicrobial activity of twenty commercial frankincense essential oil samples. South Afr. J. Bot..

[10] Silva G.L., Luft C., Lunardelli A., Amaral R.H., Melo D.A., Donadio M.V., Nunes F.B., de Azambuja M.S., Santana J.C., Moraes C.M. (2015). Antioxidant, analgesic and anti-inflammatory effects of lavender essential oil. An. Acad Bras. Cienc..

[11] Scuteri D., Hamamura K., Sakurada T., Watanabe C., Sakurada S., Morrone L.A., Rombola L., Tonin P., Bagetta G., Corasaniti M.T. (2021). Efficacy of Essential Oils in Pain: A Systematic Review and Meta-Analysis of Preclinical Evidence. Front. Pharmacol..

[12] Lehrner J., Marwinski G., Lehr S., Johren P., Deecke L. (2005). Ambient odors of orange and lavender reduce anxiety and improve mood in a dental office. Physiol Behav..

[13] Conrad P., Adams C. (2012). The effects of clinical aromatherapy for anxiety and depression in the high risk postpartum woman—A pilot study. Complementary Ther. Clin. Pract..

[14] Okano K., Kaczmarzyk J.R., Dave N., Gabrieli J.D.E., Grossman J.C. (2019). Sleep quality, duration, and consistency are associated with better academic performance in college students. NPJ Sci. Learn..

[15] Lillehei A.S., Halcon L.L., Savik K., Reis R. (2015). Effect of Inhaled Lavender and Sleep Hygiene on Self-Reported Sleep Issues: A Randomized Controlled Trial. J. Altern. Complementary Med..

[16] Faydali S., Cetinkaya F. (2018). The Effect of Aromatherapy on Sleep Quality of Elderly People Residing in a Nursing Home. Holist. Nurs. Pract..

[17] Pokajewicz K., Bialon M., Svydenko L., Fedin R., Hudz N. (2021). Chemical Composition of the Essential Oil of the New Cultivars of Lavandula angustifolia Mill. Bred in Ukraine. Molecules.

[18] Malcolm B.J., Tallian K. (2017). Essential oil of lavender in anxiety disorders: Ready for prime time?. Ment. Health Clin..

[19] Ekpenyong C.E., Akpan E.E. (2017). Use of *Cymbopogon citratus* essential oil in food preservation: Recent advances and future perspectives. Crit. Rev. Food Sci. Nutr..

[20] Costa C.A., Cury T.C., Cassettari B.O., Takahira R.K., Florio J.C., Costa M. (2013). *Citrus aurantium* L. essential oil exhibits anxiolytic-like activity mediated by 5-HT(1A)-receptors and reduces cholesterol after repeated oral treatment. BMC Complementary Altern. Med..

[21] Costa C.A., Kohn D.O., de Lima V.M., Gargano A.C., Florio J.C., Costa M. (2011). The GABAergic system contributes to the anxiolytic-like effect of essential oil from *Cymbopogon citratus* (lemongrass). J. Ethnopharmacol..

[22] Goes T.C., Ursulino F.R., Almeida-Souza T.H., Alves P.B., Teixeira-Silva F. (2015). Effect of Lemongrass Aroma on Experimental Anxiety in Humans. J. Altern. Complementary Med..

[23] Tan L.T., Lee L.H., Yin W.F., Chan C.K., Abdul Kadir H., Chan K.G., Goh B.H. (2015). Traditional Uses, Phytochemistry, and Bioactivities of *Cananga odorata* (Ylang-Ylang). Evid. Based Complementary Alternat. Med..

[24] Zhang N., Zhang L., Feng L., Yao L. (2016). The anxiolytic effect of essential oil of *Cananga odorata* exposure on mice and determination of its major active constituents. Phytomedicine.

[25] Jung D.J., Cha J.Y., Kim S.E., Ko I.G., Jee Y.S. (2013). Effects of Ylang-Ylang aroma on blood pressure and heart rate in healthy men. J. Exerc. Rehabil..

[26] Zhang N., Zhang L., Feng L., Yao L. (2018). *Cananga odorata* essential oil reverses the anxiety induced by 1-(3-chlorophenyl) piperazine through regulating the MAPK pathway and serotonin system in mice. J. Ethnopharmacol..

[27] Giang P.M., Son P.T. (2016). GC and GC-MS analysis of the fresh flower essential oil of *Cananga odorata* (Lam) Hook. f. et Th. var. fruticosa (Craib). J. Sincl. Am. J. Essent. Oils Nat. Prod..

[28] Hwang E.S., Kim H.B., Lee S., Kim M.J., Kim K.J., Han G., Han S.Y., Lee E.A., Yoon J.H., Kim D.O. (2020). Antidepressant-like effects of beta-caryophyllene on restraint plus stress-induced depression. Behav. Brain Res..

[29] Sohrabi R., Pazgoohan N., Seresht H.R., Amin B. (2017). Repeated systemic administration of the cinnamon essential oil possesses anti-anxiety and anti-depressant activities in mice. Iran. J. Basic. Med. Sci..

[30] Chen Y.F., Wang Y.W., Huang W.S., Lee M.M., Wood W.G., Leung Y.M., Tsai H.Y. (2016). Trans-Cinnamaldehyde, An Essential Oil in Cinnamon Powder, Ameliorates Cerebral Ischemia-Induced Brain Injury via Inhibition of Neuroinflammation Through Attenuation of iNOS, COX-2 Expression and NFkappa-B Signaling Pathway. Neuromolecular. Med..

[31] Iwasaki Y., Tanabe M., Kobata K., Watanabe T. (2008). TRPA1 agonists--allyl isothiocyanate and cinnamaldehyde--induce adrenaline secretion. Biosci. Biotechnol. Biochem..

[32] Alizadeh Behbahani B., Falah F., Lavi Arab F., Vasiee M., Tabatabaee Yazdi F. (2020). Chemical Composition and Antioxidant, Antimicrobial, and Antiproliferative Activities of *Cinnamomum zeylanicum* Bark Essential Oil. Evid. Based. Complementary Altern. Med..

[33] Sebei K., Sakouhi F., Herchi W., Khouja M.L., Boukhchina S. (2015). Chemical composition and antibacterial activities of seven Eucalyptus species essential oils leaves. Biol. Res..

[34] Juergens U.R. (2014). Anti-inflammatory properties of the monoterpene 1.8-cineole: Current evidence for co-medication in inflammatory airway diseases. Drug Res..

[35] Russo E.B. (2011). Taming THC: Potential cannabis synergy and phytocannabinoid-terpenoid entourage effects. Br. J. Pharmacol..

[36] Nissen L., Zatta A., Stefanini I., Grandi S., Sgorbati B., Biavati B., Monti A. (2010). Characterization and antimicrobial activity of essential oils of industrial hemp varieties (*Cannabis sativa* L.). Fitoterapia.

[37] Brett A.E., A.Webster A., Robertson D., Burnstock G., Paton J.F.R., Biaggioni I., Low P.A. (2012). Chapter 132—Acetylcholinesterase and its Inhibitors. Primer on the Autonomic Nervous System (Third Edition).

[38] Jun Y.S., Kang P., Min S.S., Lee J.M., Kim H.K., Seol G.H. (2013). Effect of eucalyptus oil inhalation on pain and inflammatory responses after total knee replacement: A randomized clinical trial. Evid. Based Complementary Altern. Med..

[39] Hsouna A.B., Touj N., Hammami I., Dridi K., Al-Ayed A.S., Hamdi N. (2019). Chemical Composition and in vivo Efficacy of the Essential Oil of *Mentha piperita* L. in the Suppression of Crown Gall Disease on Tomato Plants. J. Oleo Sci..

[40] Masomeh L., Narges M., Hassan R.A.H. (2017). Peppermint and Its Functionality: A Review. Arch. Clin. Microbiol..

[41] Umezu T. (2012). Evaluation of the effects of plant-derived essential oils on central nervous system function using discrete shuttle-type conditioned avoidance response in mice. Phytother. Res..

[42] Kennedy D., Okello E., Chazot P., Howes M.J., Ohiomokhare S., Jackson P., Haskell-Ramsay C., Khan J., Forster J., Wightman E. (2018). Volatile Terpenes and Brain Function: Investigation of the Cognitive and Mood Effects of *Mentha* × *Piperita* L. Essential Oil with In Vitro Properties Relevant to Central Nervous System Function. Nutrients.

[43] Bhadania M., Joshi H., Patel P., Kulkarni V.H. (2012). Protective effect of menthol on beta-amyloid peptide induced cognitive deficits in mice. Eur. J. Pharmacol..

[44] Schmidt E., Bail S., Buchbauer G., Stoilova I., Atanasova T., Stoyanova A., Krastanov A., Jirovetz L. (2009). Chemical composition, olfactory evaluation and antioxidant effects of essential oil from *Mentha* × *piperita*. Nat. Prod. Commun..

[45] Moss M., Hewitt S., Moss L., Wesnes K. (2008). Modulation of cognitive performance and mood by aromas of peppermint and ylang-ylang. Int. J. Neurosci..

[46] Elyemni M., Louaste B., Nechad I., Elkamli T., Bouia A., Taleb M., Chaouch M., Eloutassi N. (2019). Extraction of Essential Oils of *Rosmarinus officinalis* L. by Two Different Methods: Hydrodistillation and Microwave Assisted Hydrodistillation. Sci. World J..

[47] Villareal M.O., Ikeya A., Sasaki K., Arfa A.B., Neffati M., Isoda H. (2017). Anti-stress and neuronal cell differentiation induction effects of *Rosmarinus officinalis* L. essential oil. BMC Complementary Altern. Med..

[48] Habtemariam S. (2016). The Therapeutic Potential of Rosemary (*Rosmarinus officinalis*) Diterpenes for Alzheimer’s Disease. Evid. Based Complementary Altern. Med..

[49] Ozcan M.M., Chalchat J.C. (2008). Chemical composition and antifungal activity of rosemary (*Rosmarinus officinalis* L.) oil from Turkey. Int. J. Food Sci. Nutr..

[50] Jiang Y., Wu N., Fu Y.J., Wang W., Luo M., Zhao C.J., Zu Y.G., Liu X.L. (2011). Chemical composition and antimicrobial activity of the essential oil of Rosemary. Environ. Toxicol. Pharmacol..

[51] Khedher M.R.B., Khedher S.B., Chaieb I., Tounsi S., Hammami M. (2017). Chemical composition and biological activities of *Salvia officinalis* essential oil from Tunisia. EXCLI J..

[52] Moss M., Rouse M., Moss L. (2014). Aromas of Salvia Species Enhance Everyday Prospective Memory Performance in Healthy Young Adults. Adv. Chem. Eng. Sci..

[53] Lopresti A.L. (2017). Salvia (Sage): A Review of its Potential Cognitive-Enhancing and Protective Effects. Drugs R D.

[54] Akhondzadeh S., Noroozian M., Mohammadi M., Ohadinia S., Jamshidi A.H., Khani M. (2003). *Salvia officinalis* extract in the treatment of patients with mild to moderate Alzheimer’s disease: A double blind, randomized and placebo-controlled trial. J. Clin. Pharm. Ther..

[55] Craft J.D., Satyal P., Setzer W.N. (2017). The Chemotaxonomy of Common Sage (*Salvia officinalis*) Based on the Volatile Constituents. Medicines.

[56] Braun N.A., Sim S., Kohlenberg B., Lawrence B.M. (2014). Hawaiian sandalwood: Oil composition of *Santalum paniculatum* and comparison with other sandal species. Nat. Prod. Commun..

[57] Safwat Y.N., Elsayed M.M. (2020). Sandalwood oil neuroprotective effects on middle cerebral artery occlusion model of ischemic brain stroke. Farmacogn. Mag..

[58] Misra B.B., Dey S. (2013). Biological Activities of East Indian Sandalwood Tree, Santalum album. PeerJ.

[59] . Misra B.B., Dey S. (2013). Evaluation of in vivo anti-hyperglycemic and antioxidant potentials of alpha-santalol and sandalwood oil. Phytomedicine.

[60] Hoferl M., Hutter C., Buchbauer G. (2016). A Pilot Study on the Physiological Effects of Three Essential Oils in Humans. Nat. Prod. Commun..

[61] Kusuma H.S., Mahfud M. (2016). Chemical composition of essential oil of Indonesia sandalwood extracted by microwave-assisted hydrodistillation. AIP Conf. Proc..

[62] Moss M., Cook J., Wesnes K., Duckett P. (2003). Aromas of rosemary and lavender essential oils differentially affect cognition and mood in healthy adults. Int. J. Neurosci..

[63] Hadi N., Hanid A.A. (2011). Lavender Essence for Post-cesarean pain. Pak. J. Biol. Sci..

[64] Sayorwan W., Ruangrungsi N., Piriyapunyporn T., Hongratanaworakit T., Kotchabhakdi N., Siripornpanich V. (2013). Effects of inhaled rosemary oil on subjective feelings and activities of the nervous system. Sci. Pharm..

[65] Moss L., Rouse M., Wesnes K.A., Moss M. (2010). Differential effects of the aromas of *Salvia* species on memory and mood. Hum. Psychopharmacol..

[66] Boehm K., Bussing A., Ostermann T. (2012). Aromatherapy as an adjuvant treatment in cancer care--a descriptive systematic review. Afr. J. Tradit. Complementary Altern. Med..

[67] Lakhan S.E., Sheafer H., Tepper D. (2016). The Effectiveness of Aromatherapy in Reducing Pain: A Systematic Review and Meta-Analysis. Pain Res. Treat..

[68] Yang S., Chang M.C. (2019). Chronic Pain: Structural and Functional Changes in Brain Structures and Associated Negative Affective States. Int. J. Mol. Sci..

[69] Wang Z.J., Heinbockel T. (2018). Essential Oils and Their Constituents Targeting the GABAergic System and Sodium Channels as Treatment of Neurological Diseases. Molecules.

[70] WHO Depression. https://www.who.int/news-room/fact-sheets/detail/depression.

[71] Thibaut F. (2017). Anxiety disorders: A review of current literature. Dialogues Clin. Neurosci..

[72] Ogata K., Ataka K., Suzuki H., Yagi T., Okawa A., Fukumoto T., Zhang B., Nakata M., Yada T., Asakawa A. (2020). Lavender Oil Reduces Depressive Mood in Healthy Individuals and Enhances the Activity of Single Oxytocin Neurons of the Hypothalamus Isolated from Mice: A Preliminary Study. Evid. Based Complementary Altern. Med..

[73] WHO Dementia. https://www.who.int/news-room/fact-sheets/detail/dementia.

[74] National Institute of Ageing Treatment of Alzeimers Disease. https://www.nia.nih.gov/health/how-alzheimers-disease-treated.

[75] Santarsieri D., Schwartz T.L. (2015). Antidepressant efficacy and side-effect burden: A quick guide for clinicians. Drugs Context.

[76] Filiptsova O.V., Gazzavi-Rogozina L.V., Timoshyna I.A., Naboka O.I., Dyomina Y.V., Ochkur A.V. (2018). The effect of the essential oils of lavender and rosemary on the human short-term memory. Alex. J. Med..

[77] Page M.J., McKenzie J.E., Bossuyt P.M., Boutron I., Hoffmann T.C., Mulrow C.D., Shamseer L., Tetzlaff J.M., Moher D. (2021). Updating guidance for reporting systematic reviews: Development of the PRISMA 2020 statement. J. Clin. Epidemiol..

[78] Page M.J., McKenzie J.E., Bossuyt P.M., Boutron I., Hoffmann T.C., Mulrow C.D., Shamseer L., Tetzlaff J.M., Akl E.A., Brennan S.E. (2021). The PRISMA 2020 statement: An updated guideline for reporting systematic reviews. J. Clin. Epidemiol..

[79] Dagli N., Dagli R., Mahmoud R.S., Baroudi K. (2015). Essential oils, their therapeutic properties, and implication in dentistry: A review. J. Int. Soc. Prev. Community Dent..

[80] Sá R.D.C.D.S.E., Lima T.C., da Nóbrega F.R., Brito A.E.M.D., de Sousa D.P. (2017). Analgesic-Like Activity of Essential Oil Constituents: An Update. Int. J. Mol. Sci..

[81] Pramod K., Ansari S.H., Ali J. (2010). Eugenol: A Natural Compound with Versatile Pharmacological Actions. Nat. Prod. Commun..

[82] Chung G., Rhee J.N., Jung S.J., Kim J.S., Oh S.B. (2008). Modulation of CaV2.3 calcium channel currents by eugenol. J. Dent. Res..

[83] Cho J.S., Kim T.H., Lim J.M., Song J.H. (2008). Effects of eugenol on Na+ currents in rat dorsal root ganglion neurons. Brain Res..

[84] Aoshima H., Hamamoto K. (1999). Potentiation of GABAA Receptors Expressed in Xenopus Oocytes by Perfume and Phytoncid. Biosci. Biotechnol. Biochem..

[85] Dal Bo W., Luiz A.P., Martins D.F., Mazzardo-Martins L., Santos A.R. (2013). Eugenol reduces acute pain in mice by modulating the glutamatergic and tumor necrosis factor alpha (TNF-alpha) pathways. Fundam. Clin. Pharmacol..

[86] Dhiman P., Malik N., Khatkar A. (2019). Lead optimization for promising monoamine oxidase inhibitor from eugenol for the treatment of neurological disorder: Synthesis and in silico based study. BMC Chem..

[87] Holanda Pinto S.A., Pinto L.M., Guedes M.A., Cunha G.M., Chaves M.H., Santos F.A., Rao V.S. (2008). Antinoceptive effect of triterpenoid alpha,beta-amyrin in rats on orofacial pain induced by formalin and capsaicin. Phytomedicine.

[88] Venâncio A.M., Marchioro M., Estavam C.S., Melo M.S., Santana M.T., Onofre A.S.C., Guimarães A.G., Oliveira M.G.B., Alves P.B., Pimentel H.D.C. (2011). *Ocimum basilicum* leaf essential oil and (-)-linalool reduce orofacial nociception in rodents: A behavioral and electrophysiological approach. Braz. J. Farmacogn..

[89] Andersen P., Bliss T.V., Skrede K.K. (1971). Unit analysis of hippocampal polulation spikes. Exp. Brain Res..

[90] Peana A.T., De Montis M.G., Nieddu E., Spano M.T., D’Aquila P.S., Pippia P. (2004). Profile of spinal and supra-spinal antinociception of (-)-linalool. Eur. J. Pharmacol..

[91] Peana A.T., D’Aquila P.S., Chessa M.L., Moretti M.D., Serra G., Pippia P. (2003). (-)-Linalool produces antinociception in two experimental models of pain. Eur. J. Pharmacol..

[92] Tashiro S., Yamaguchi R., Ishikawa S., Sakurai T., Kajiya K., Kanmura Y., Kuwaki T., Kashiwadani H. (2016). Odour-induced analgesia mediated by hypothalamic orexin neurons in mice. Sci. Rep..

[93] Johnson S.A., Rodriguez D., Allred K. (2020). A Systematic Review of Essential Oils and the Endocannabinoid System: A Connection Worthy of Further Exploration. Evid. Based Complementary Altern. Med..

[94] Anxiety and Depression Association of America Anxiety, Facts and Statistics. https://adaa.org/understanding-anxiety/facts-statistics.

[95] MayoClinic Anxiety Disorders, Diagnosis and Treatment. https://www.mayoclinic.org/diseases-conditions/anxiety/diagnosis-treatment/drc-20350967.

[96] NIH Depression. https://www.nimh.nih.gov/health/topics/depression/.

[97] MayoClinic Depression (Major Depressive Disorder). https://www.mayoclinic.org/diseases-conditions/depression/symptoms-causes/syc-20356007.

[98] Machado K.D.C., Islam M.T., Ali E.S., Rouf R., Uddin S.J., Dev S., Shilpi J.A., Shill M.C., Reza H.M., Das A.K. (2018). A systematic review on the neuroprotective perspectives of beta-caryophyllene. Phytother. Res..

[99] Galaj E., Xi Z.X. (2019). Potential of Cannabinoid Receptor Ligands as Treatment for Substance Use Disorders. CNS Drugs.

[100] Johnson W.G. (2000). Late-onset neurodegenerative diseases--the role of protein insolubility. J. Anat..

[101] Gertsch J. (2008). Anti-inflammatory cannabinoids in diet: Towards a better understanding of CB(2) receptor action?. Commun. Integr. Biol.

[102] Paula-Freire L.I., Andersen M.L., Gama V.S., Molska G.R., Carlini E.L. (2014). The oral administration of trans-caryophyllene attenuates acute and chronic pain in mice. Phytomedicine.

[103] Katsuyama S., Mizoguchi H., Kuwahata H., Komatsu T., Nagaoka K., Nakamura H., Bagetta G., Sakurada T., Sakurada S. (2013). Involvement of peripheral cannabinoid and opioid receptors in beta-caryophyllene-induced antinociception. Eur. J. Pain.

[104] Association A.S. What Is Alzheimer’s Disease?. https://www.alz.org/alzheimers-dementia/what-is-alzheimers.

[105] National Institute on Ageing Alzheimer’s Disease Fact Sheet. https://www.nia.nih.gov/health/alzheimers-disease-fact-sheet.

[106] Reiman E.M., Langbaum J.B., Fleisher A.S., Caselli R.J., Chen K., Ayutyanont N., Quiroz Y.T., Kosik K.S., Lopera F., Tariot P.N. (2011). Alzheimer’s Prevention Initiative: A plan to accelerate the evaluation of presymptomatic treatments. J. Alzheimers Dis..

[107] Lawther B.K., Kumar S., Krovvidi H. (2011). Blood–brain barrier. Continuing Education in Anaesthesia, Critical Care and Pain.

[108] Wong H.L., Bendayan R., Rauth A.M., Li Y., Wu X.Y. (2007). Chemotherapy with anticancer drugs encapsulated in solid lipid nanoparticles. Adv. Drug Deliv. Rev..

[109] Palmerston Mendes L., Pan J., Torchilin V.P. (2017). Dendrimers as Nanocarriers for Nucleic Acid and Drug Delivery in Cancer Therapy. Molecules.

[110] Smith D.K. (2012). Designing Dendrimers.

[111] Xu L., Zhang H., Wu Y. (2014). Dendrimer advances for the central nervous system delivery of therapeutics. ACS Chem. Neurosci..

[112] Menjoge A.R., Kannan R.M., Tomalia D.A. (2010). Dendrimer-based drug and imaging conjugates: Design considerations for nanomedical applications. Drug Discov. Today.

[113] Huang R.Q., Qu Y.H., Ke W.L., Zhu J.H., Pei Y.Y., Jiang C. (2007). Efficient gene delivery targeted to the brain using a transferrin-conjugated polyethyleneglycol-modified polyamidoamine dendrimer. FASEB J..

[114] Huang R., Ke W., Han L., Li J., Liu S., Jiang C. (2011). Targeted delivery of chlorotoxin-modified DNA-loaded nanoparticles to glioma via intravenous administration. Biomaterials.

[115] He H., Li Y., Jia X.R., Du J., Ying X., Lu W.L., Lou J.N., Wei Y. (2011). PEGylated Poly(amidoamine) dendrimer-based dual-targeting carrier for treating brain tumors. Biomaterials.

[116] Liu Y., Huang R., Han L., Ke W., Shao K., Ye L., Lou J., Jiang C. (2009). Brain-targeting gene delivery and cellular internalization mechanisms for modified rabies virus glycoprotein RVG29 nanoparticles. Biomaterials.

[117] Tagde P., Tagde P., Tagde S., Bhattacharya T., Garg V., Akter R., Rahman M.H., Najda A., Albadrani G.M., Sayed A.A. (2021). Natural bioactive molecules: An alternative approach to the treatment and control of glioblastoma multiforme. Biomed. Pharmacother..

[118] Sharma A., Sharma R., Zhang Z., Liaw K., Kambhampati S.P., Porterfield J.E., Lin K.C., DeRidder L.B., Kannan S., Kannan R.M. (2020). Dense hydroxyl polyethylene glycol dendrimer targets activated glia in multiple CNS disorders. Sci. Adv..

[119] Sultana F., Manirujjaman, Imran-Ul-Haque M., Arafat M., Sharmin S. (2013). An Overview of Nanogel Drug Delivery System. J. Appl. Pharm. Sci..

[120] Kabanov A.V., Gendelman H.E. (2007). Nanomedicine in the diagnosis and therapy of neurodegenerative disorders. Prog. Polym. Sci..

[121] Vinogradov S. (2004). The second annual symposium on nanomedicine and drug delivery: Exploring recent developments and assessing major advances. 19-20 August 2004, Polytechnic University, Brooklyn, NY, USA. Expert. Opin. Drug Deliv..

[122] Vinogradov S.V., Batrakova E.V., Kabanov A.V. (2004). Nanogels for oligonucleotide delivery to the brain. Bioconjug. Chem..

[123] Azadi A., Rouini M.R., Hamidi M. (2015). Neuropharmacokinetic evaluation of methotrexate-loaded chitosan nanogels. Int. J. Biol. Macromol..

[124] Gulyaev A.E., Gelperina S.E., Skidan I.N., Antropov A.S., Kivman G.Y., Kreuter J. (1999). Significant transport of doxorubicin into the brain with polysorbate 80-coated nanoparticles. Pharm. Res..

[125] Kumar A., Chaudhary R.K., Singh R., Singh S.P., Wang S.Y., Hoe Z.Y., Pan C.T., Shiue Y.L., Wei D.Q., Kaushik A.C. (2020). Nanotheranostic Applications for Detection and Targeting Neurodegenerative Diseases. Front. Neurosci..

[126] Goyal K., Koul V., Singh Y., Anand A. (2014). Targeted drug delivery to central nervous system (CNS) for the treatment of neurodegenerative disorders: Trends and advances. Cent. Nerv. Syst. Agents Med. Chem..

[127] Saeedi M., Eslamifar M., Khezri K., Dizaj S.M. (2019). Applications of nanotechnology in drug delivery to the central nervous system. Biomed. Pharmacother..

[128] Kafa H., Wang J.T., Rubio N., Venner K., Anderson G., Pach E., Ballesteros B., Preston J.E., Abbott N.J., Al-Jamal K.T. (2015). The interaction of carbon nanotubes with an in vitro blood-brain barrier model and mouse brain in vivo. Biomaterials.

[129] Aziz Z.A.A., Nasir H.M., Ahmad A., Setapar S.H.M., Ahmad H., Noor M.H.M., Rafatullah M., Khatoon A., Kausar M.A., Ahmad I. (2019). Enrichment of Eucalyptus oil nanoemulsion by micellar nanotechnology: Transdermal analgesic activity using hot plate test in rats’ assay. Sci. Rep..

[130] Scuteri D., Cassano R., Trombino S., Russo R., Mizoguchi H., Watanabe C., Hamamura K., Katsuyama S., Komatsu T., Morrone L.A. (2021). Development and Translation of NanoBEO, a Nanotechnology-Based Delivery System of Bergamot Essential Oil Deprived of Furocumarins, in the Control of Agitation in Severe Dementia. Pharmaceutics.

[131] Gaude T.T., Soares G.A.B.E., Priolkar R.N.S., Biradar B., Mamledesai1 S. (2017). Synthesis of 4-hydroxy-1-(phenyl/methyl)-3-[3-(substituted amino)-2-nitropropanoyl] quinolin-2(1H)-ones as an antimicrobial andantitubercular agents. Indian J. Heterocycl. Chem..

[132] Sharifi-Rad J., Sureda A., Tenore G.C., Daglia M., Sharifi-Rad M., Valussi M., Tundis R., Sharifi-Rad M., Loizzo M.R., Ademiluyi A.O. (2017). Biological Activities of Essential Oils: From Plant Chemoecology to Traditional Healing Systems. Molecules.

[133] Dosoky N.S., Setzer W.N. (2021). Maternal Reproductive Toxicity of Some Essential Oils and Their Constituents. Int. J. Mol. Sci..

[134] Conlon P.M., Haack K.M., Rodgers N.J., Dion L.J., Cambern K.L., Rohlik G.M., Nelson D.E., Barry T.A., Ayres S.J., Cutshall S.M. (2017). Introducing Essential Oils into Pediatric and Other Practices at an Academic Medical Center. J. Holist. Nurs..

[135] de Matos S.P., Teixeira H.F., de Lima A.A.N., Veiga-Junior V.F., Koester L.S. (2019). Essential Oils and Isolated Terpenes in Nanosystems Designed for Topical Administration: A Review. Biomolecules.

[136] Sapra B., Jain S., Tiwary A.K. (2008). Percutaneous permeation enhancement by terpenes: Mechanistic view. AAPS J..

[137] Lalko J., Api A.M. (2006). Investigation of the dermal sensitization potential of various essential oils in the local lymph node assay. Food Chem. Toxicol..

[138] Opdyke D.L.J. (1976). Inhibition of sensitization reactions induced by certain aldehydes. Food Cosmet. Toxicol..

[139] Nilsson A.M., Gafvert E., Salvador L., Luthman K., Bruze M., Gruvberger B., Nilsson J.L., Karlberg A.T. (2001). Mechanism of the antigen formation of carvone and related alpha, beta-unsaturated ketones. Contact Dermat..

[140] Nilsson A.M., Jonsson C., Luthman K., Nilsson J.L., Karlberg A.T. (2004). Inhibition of the sensitizing effect of carvone by the addition of non-allergenic compounds. Acta Derm. Venereol..

[141] Karlberg A.T., Nilsson A.M., Luthman K., Nilsson J.L. (2001). Structural analogues inhibit the sensitizing capacity of carvone. Acta Derm. Venereol..

[142] Basketter D. (2000). Quenching: Fact or fiction?. Contact Dermat..

[143] Marturano V., Bizzarro V., De Luise A., Calarco A., Ambrogi V., Giamberini M., Tylkowski B., Cerruti P. (2018). Essential oils as solvents and core materials for the preparation of photo-responsive polymer nanocapsules. Nano Res..

[144] Mashwani Z.U., Khan M.A., Khan T., Nadhman A. (2016). Applications of plant terpenoids in the synthesis of colloidal silver nanoparticles. Adv. Colloid Interface Sci..

[145] Perino-Issartier S., Ginies C., Cravotto G., Chemat F. (2013). A comparison of essential oils obtained from lavandin via different extraction processes: Ultrasound, microwave, turbohydrodistillation, steam and hydrodistillation. J. Chromatogr. A.

[146] Anastas P.T., Kirchhoff M.M. (2002). Origins, current status, and future challenges of green chemistry. Acc. Chem. Res..

[147] Tucker J.L. (2006). Green Chemistry, a Pharmaceutical Perspective. Org. Process. Res. Dev..

[148] Patel K.D., Davison J.S., Pittman Q.J., Sharkey K.A. (2010). Cannabinoid CB(2) receptors in health and disease. Curr. Med. Chem..

[149] Cassano T., Calcagnini S., Pace L., De Marco F., Romano A., Gaetani S. (2017). Cannabinoid Receptor 2 Signaling in Neurodegenerative Disorders: From Pathogenesis to a Promising Therapeutic Target. Front. Neurosci..

[150] Ramirez B.G., Blazquez C., Gomez del Pulgar T., Guzman M., de Ceballos M.L. (2005). Prevention of Alzheimer’s disease pathology by cannabinoids: Neuroprotection mediated by blockade of microglial activation. J. Neurosci..

[151] Farooqui A.A. (2019). Potential Treatment Strategies for the Treatment of Dementia With Chinese Medicinal Plants. Molecular Mechanisms of Dementia.

[152] Koppel J., Bradshaw H., Goldberg T.E., Khalili H., Marambaud P., Walker M.J., Pazos M., Gordon M.L., Christen E., Davies P. (2009). Endocannabinoids in Alzheimer’s disease and their impact on normative cognitive performance: A case-control and cohort study. Lipids Health Dis..

[153] Sharma C., Sadek B., Goyal S.N., Sinha S., Kamal M.A., Ojha S. (2015). Small Molecules from Nature Targeting G-Protein Coupled Cannabinoid Receptors: Potential Leads for Drug Discovery and Development. Evid. Based Complementary Altern. Med..

[154] Gulluni N., Re T., Loiacono I., Lanzo G., Gori L., Macchi C., Epifani F., Bragazzi N., Firenzuoli F. (2018). Cannabis Essential Oil: A Preliminary Study for the Evaluation of the Brain Effects. Evid. Based Complementary Altern. Med..

[155] Pacher P., Kunos G. (2013). Modulating the endocannabinoid system in human health and disease--successes and failures. FEBS J..

[156] Shepard B.D., Pluznick J.L. (2016). How does your kidney smell? Emerging roles for olfactory receptors in renal function. Pediatric Nephrol..

